# The influence of gender and temephos exposure on community participation in dengue prevention: a compartmental mathematical model

**DOI:** 10.1186/s12879-024-09341-w

**Published:** 2024-05-02

**Authors:** Víctor Manuel Alvarado-Castro, Cruz Vargas-De-León, Sergio Paredes-Solis, Alian Li-Martin, Elizabeth Nava-Aguilera, Arcadio Morales-Pérez, José Legorreta-Soberanis, Belén Madeline Sánchez-Gervacio, Anne Cockcroft, Neil Andersson

**Affiliations:** 1 Centro de Investigación de Enfermedades Tropicales, Universidad Autónoma de Guerrero, Acapulco, Guerrero C.P. 39640 México; 2 División de Investigación, Hospital Juárez de México, Ciudad de Mexico, 07760 México; 3 Sección de Estudios de Posgrado, Escuela Superior de Medicina, Instituto Politécnico Nacional, Ciudad de Mexico, 11340 México; 4 Facultad de Matemáticas, Universidad Autónoma de Guerrero, Chilpancingo, Guerrero C.P. 39017 México; 5 Department of Family Medicine, McGill University, Montreal, P.C. H3S 1Z1 Canada

**Keywords:** Dengue, *Aedes aegypti*, Community participation, Compartmental model, Temephos

## Abstract

**Background:**

The use of temephos, the most common intervention for the chemical control of *Aedes aegypti* over the last half century, has disappointing results in control of the infection. The footprint of *Aedes* and the diseases it carries have spread relentlessly despite massive volumes of temephos. Recent advances in community participation show this might be more effective and sustainable for the control of the dengue vector.

**Methods:**

Using data from the *Camino Verde* cluster randomized controlled trial, a compartmental mathematical model examines the dynamics of dengue infection with different levels of community participation, taking account of gender of respondent and exposure to temephos.

**Results:**

Simulation of dengue endemicity showed community participation affected the basic reproductive number of infected people. The greatest short-term effect, in terms of people infected with the virus, was the combination of temephos intervention and community participation. There was no evidence of a protective effect of temephos 220 days after the onset of the spread of dengue.

**Conclusions:**

Male responses about community participation did not significantly affect modelled numbers of infected people and infectious mosquitoes. Our model suggests that, in the long term, community participation alone may have the best results. Adding temephos to community participation does not improve the effect of community participation alone.

**Supplementary Information:**

The online version contains supplementary material available at 10.1186/s12879-024-09341-w.

## Background

Control of the *Aedes aegypti* is the cornerstone of public health programs to prevent transmission [1, 2] of at least four systemic viral diseases carried by the mosquito, dengue fever [3], zika [4], chikungunya [5] and yellow fever [6]. For most of the last century, temephos has been at the centre of chemical control of the vector [7, 8]. Limited effectiveness may be related to the small window of chemical activity and increasing resistance to temephos [8, 9].

Several studies show low effectiveness of the pesticide on the dengue transmission, for reasons including larval resistance to temephos [8, 10, 11]. Aside from its inconclusive impact on dengue transmission, widespread use of temephos may produce a sense of “being protected” against dengue that could discourage participation in prevention activities [12]. Recent approaches to community participation seem to be more sustainable and effective [13–15]. A randomised controlled trial in Mexico and Nicaragua demonstrated that community participation adds effectiveness to government dengue control programs [12]. The same effect was not achieved across all intervention sites in that trial, however, and gender issues and social capital might influence the impact of community participation [16].

Many mathematical models of infection transmission rely on the 1927 proposal of Kermack and McKendrick. Summarised by the acronym SIR, this divides the population in three compartments: **s**usceptible, without the infection but on risk of being infected; **i**nfected, with the disease and be able to spread it; **r**ecovered, those who recovers from the disease and have permanent immunity against the disease. In the case of dengue, several SIR models attempted to explain the dynamics of different dengue virus serotypes [17–20] and to analyse the effect of vector control strategies [21–23]. Compartmental models usually precede optimum control models, [24–26] used to analyse combination of control mechanisms for *Aedes aegypti*, to quantify the impact of different strategies while minimize or maximize the effect of each intervention in individual or in combination [27].

A previous study showed gender of respondents affected the results chain for dengue prevention behaviour [16] but we could not find published of mathematical models of gender and temephos on community participation in dengue vector control. We present a compartmental mathematical model that helps to explain the dynamics of the spread of virus dengue infection, considering different levels of community participation by gender and temephos exposure.

## Methods

### Formulation of the model

We used data from impact measurement survey of the Mexico arm of the *Camino Verde* [12] trial to explore community participation according to male and female respondents, and to explore the linkages between participation and temephos exposure. For this proposal, we understand community participation as specific groups with shared needs living in a defined geographic area actively pursue identification of their needs, take decisions and establish mechanisms to meet these needs [28]. The effect of community participation in our approach is the difference in vector indices between the intervention and control groups in the *Camino Verde* trial. We adapted the modelling approach of several authors [17, 18, 22, 25, 29, 30]. In formulation of the model, we assumed the infection is produced by only one serotype of dengue virus and that the human and *Aedes aegypti* populations are divided into compartments.

The compartments in which the populations are divided are susceptible, infectious and recovered. The dynamics for movement between these compartments are the following:

i) The human population is assumed to be constant with size equal to *N*_*h*_, with birth and death rate constant equal to *μ*_*h*_. The population is divided in susceptible, infected and recovered classes, denoted by ${\overline{S} }_{h}$, ${\overline{I} }_{h}$, and ${\overline{R} }_{h}$, respectively, with ${\overline{S} }_{h}+{\overline{I} }_{h}+{\overline{R} }_{h}={N}_{h}$. Dengue transmission is sustained by the flows between humans and mosquito compartments. The human susceptible population is decreased following the infection force rate, which can be acquired via effective contact with an exposed or infective vector at a rate
  1 $${\tau }_{h}=\alpha {\beta }_{vh}\left(1-C\left(t\right)\right)\frac{{\overline{I} }_{v}}{{N}_{h}},$$

where *α* is the biting rate per susceptible vector, *β*_*vh*_ is the transmission probability from an infective vector (${\overline{I} }_{v}$) to a susceptible human (${\overline{S} }_{h}$), and *C(t)* is the effort made by men and women in the control of *Aedes aegypti* through community participation. Infectious humans recover at a constant rate *ρ*.

ii) Following Moulay [31], a stage structured model describes the vector population dynamics, which consists of three stages: egg (*E*), larva (*L*) and pupa (*P*). We differentiate between eggs (*E*) and aquatic stages (*L* and *P*), because they respond differently to the control measures. In order to study the influence of the use of temephos and community participation, the breeding sites of *Aedes aegypti* were divided in targeted by themephos household containers (*thc*) -- tanks, drums, water tanks and cisterns -- and non-target household containers (*nthc*) that periodically renew the water or containers that can be considered as garbage to eliminate them. We assume that the average number of eggs is proportional to the number of female mosquitoes that will result, and that the availability of nutrients and space is different in each type of containers (*thc* and *nthc*); similarly, the number of eggs is proportional to the number of larvae and pupae.

The adult female mosquitoes *M* is divided in two compartments: susceptible and infective, whose are denoted by ${\overline{S} }_{v}$ and ${\overline{I} }_{v}$. The vector susceptible population is decreased following infection, which can be acquired via effective contact with an infected human at a rate

2 $${\tau }_{v}=\alpha {\beta }_{hv}\left(1-C\left(t\right)\right)\frac{{\overline{I} }_{h}}{{N}_{h}},$$

where *β*_*hv*_ is the transmission probability from an infectious human (${\overline{I} }_{h}$) to a susceptible vector (${\overline{S} }_{v}$).

Our model represents dengue transmission dynamics under the same assumptions as the Camino Verde trial: human and mosquito populations are mixed homogeneously, meaning each mosquito has the same probability of biting any individual in the population; since the outbreaks are relatively short, the population can be considered constant; reducing the mosquito population will reduce dengue cases; a single serotype is implicated in the outbreaks (allowing modelling of reduction of the susceptible population).

The entomological parameters of vector dengue, for each type of container *i* = {*thc,nthc*}, are the per capita oviposition rate *θ*_*i*_, which is the average number of eggs by female mosquito; the transition rate from egg to larva *ε*_*i*_; the transition rates from larva to pupa *λ*_*i*_ and pupa to adult *γ*_*i*_; mortality rates of eggs, larvas, pupas and mosquitoes, *μ*_*E*_, *μ*_*L*_, *μ*_*P*_, *μ*_*v*_, respectively.

**Fig. 1:**
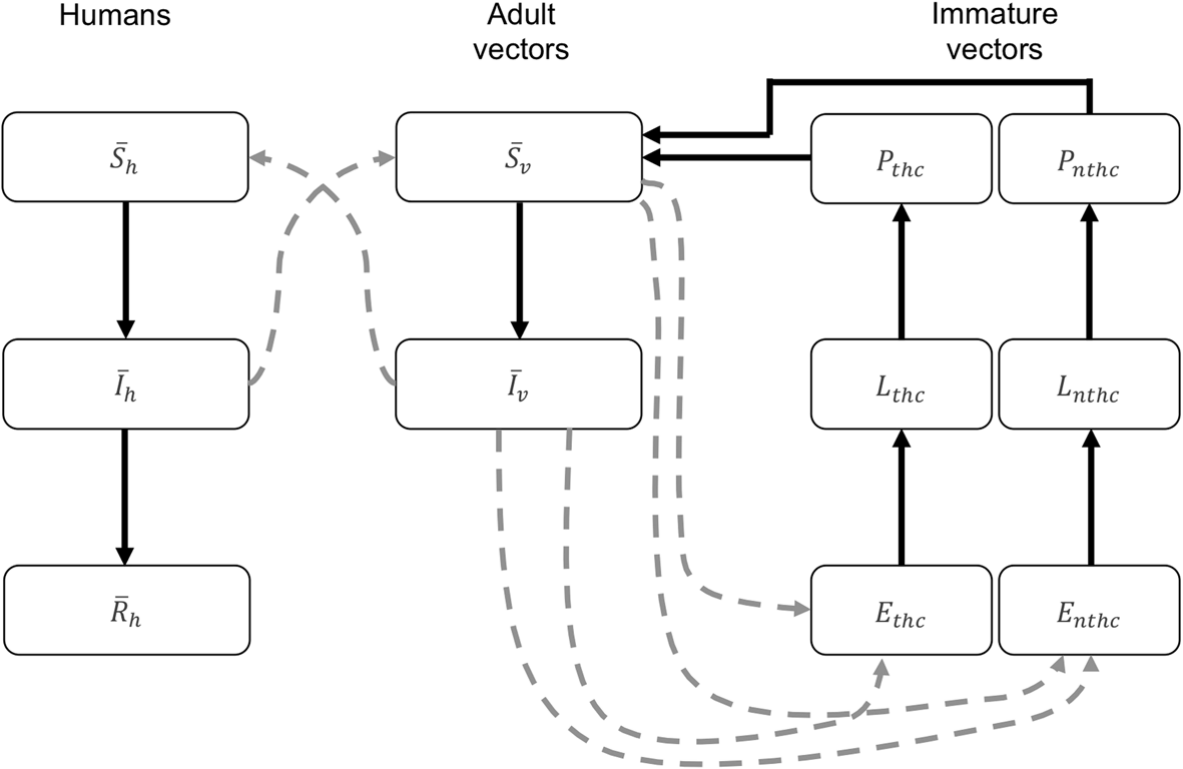
Schematic of the vector dengue model with development stages of vectors for each type of container (*thc* and *nthc*); dash lines represent infections

From the above assumptions, the model is given by the following system of differential equations:

3 $$\begin{array}{l}\frac{d{\overline{S} }_{h}}{dt}={\mu }_{h}\left({N}_{h}-{\overline{S} }_{h}\right)-{\tau }_{h}{\overline{S} }_{h}\\ \frac{d{\overline{I} }_{h}}{dt}={\tau }_{h}{\overline{S} }_{h}-\left(\rho +{\mu }_{h}\right){\overline{I} }_{h}\\ \frac{d{\overline{R} }_{h}}{dt}=\rho {\overline{I} }_{h}-{\mu }_{h}{\overline{R} }_{h}\\ \frac{d{\overline{S} }_{v}}{dt}={\gamma }_{thc}\left(t\right){P}_{thc}+{{\gamma }_{nthc}\left(t\right)P}_{nthc}-{\tau }_{v}{\overline{S} }_{v}-\left({\mu }_{v}+C\left(t\right)\right){\overline{S} }_{v}\\ \frac{d{\overline{I} }_{v}}{dt}={\tau }_{v}{\overline{S} }_{v}-\left({\mu }_{v}+C\left(t\right)\right){\overline{I} }_{v}\\ \frac{d{E}_{thc}}{dt}={\theta }_{thc}\left(1-\frac{{E}_{thc}}{{E}_{thc}^{{\text{max}}}}\right)\left({{\overline{S} }_{v}+\overline{I} }_{v}\right)-{\varepsilon }_{thc}{E}_{thc}-\left({\mu }_{E}+C\left(t\right)\right){E}_{thc}\\ \frac{d{E}_{nthc}}{dt}={\theta }_{nthc}\left(1-\frac{{E}_{nthc}}{{E}_{nthc}^{{\text{max}}}}\right)\left({{\overline{S} }_{v}+\overline{I} }_{v}\right)-{\varepsilon }_{nthc}{E}_{nthc}-\left({\mu }_{E}+C\left(t\right)\right){E}_{nthc}\\ \frac{d{L}_{thc}}{dt}={\varepsilon }_{thc}\left(1-\frac{{L}_{thc}}{{L}_{thc}^{{\text{max}}}}\right){E}_{thc}-{\lambda }_{thc}{L}_{thc}-\left({\mu }_{L}+A+C\left(t\right)\right){L}_{thc}\\ \frac{d{L}_{nthc}}{dt}={\varepsilon }_{nthc}\left(1-\frac{{L}_{nthc}}{{L}_{nthc}^{{\text{max}}}}\right){E}_{nthc}-{\lambda }_{nthc}{L}_{nthc}-\left({\mu }_{L}+C\left(t\right)\right){L}_{nthc}\\ \frac{d{P}_{thc}}{dt}={\lambda }_{thc}{L}_{thc}-{\gamma }_{thc}\left(t\right){P}_{thc}-\left({\mu }_{P}+C\left(t\right)\right){P}_{thc}\\ \frac{d{P}_{nthc}}{dt}={\lambda }_{nthc}{L}_{nthc}-{\gamma }_{nthc}\left(t\right){P}_{nthc}-\left({\mu }_{P}+C\left(t\right)\right){P}_{nthc}\end{array}$$

where *τ*_*h*_ and *τ*_*v*_ are given by (1) and (2), respectively; *A* is the effect produced by temephos in the target household containers. A schematic of the model is shown in Fig. 1. The parameters are all strict positive constants described in Table 1.

**Table 1:** Description and baseline values/range of parameters of model

Parameter	Description	Baseline value/range	Sources
*μ*_*h*_	Natural birth and mortality rate in humans	$\frac{1}{\left(73\times 365\right)}{day}^{-1}$	[32]
*α*	Average number of mosquito bites	[0.3, 1] *day*^*−1*^	[19, 26]
*β*_*vh*_	Probability of transmission of infection from an infective vector to a susceptible human	[0.1, 0.75] *day*^*−1*^	[19, 26]
*β*_*hv*_	Probability of transmission of infection from an infected human to a susceptible vector	[0.1, 0.75] *day*^*−1*^	[19, 26]
*ρ*	Recover rate for human	[0.10, 0.25] *day*^*−1*^	[19, 21, 26]
*μ*_*v*_	Natural mortality rate of vectors	[0.03, 0.125] *day*^*−1*^	[2, 26]
*θ*_*thc*_	Numbers of eggs in target household containers	[1, 6] *day*^*−1*^	[25, 33]
*θ*_*nthc*_	Numbers of eggs in no-target household containers	[1, 6]* day*^*−1*^	[25, 31]
${E}_{thc}^{{\text{max}}}$	Carrying capacity for eggs in target household containers	[10^3^, 10^6^] *day*^*−1*^	[25, 31]
${E}_{nthc}^{{\text{max}}}$	Carrying capacity for eggs in non-target household containers	[10^3^, 10^6^] *day*^*−1*^	[25, 31]
*ε*_*thc*_	Transfer rate from eggs to larvae in target household containers	0.7 *day*^*−1*^	[25, 31]
*ε*_*nthc*_	Transfer rate from eggs to larvae in non-target household containers	0.7 *day*^*−1*^	[25, 31]
*μ*_*E*_	Eggs death rate per day	[0.2, 0.4] *day*^*−1*^	[22, 31]
${L}_{thc}^{{\text{max}}}$	Carrying capacity for larvae in target household containers	[5 × 10^2^, 5 × 10^5^] *day*^*−1*^	[25, 31]
${L}_{nthc}^{{\text{max}}}$	Carrying capacity for larvae in no-target household containers	[5 × 10^2^, 5 × 10^5^] *day*^*−1*^	[25, 31]
*λ*_*thc*_	Transfer rate from larvae to pupae in target household containers	0.5 *day*^*−1*^	[25, 31]
*λ*_*nthc*_	Transfer rate from larvae to pupae in no-target household containers	0.5 *day*^*−1*^	[25, 31]
*μ*_*L*_	Larvae death rate	[0.2, 0.4] *day*^*−1*^	[31]
*μ*_*P*_	Pupae death rate	0.4 *day*^*−1*^	[25]
γ_0_	Average reproduction maturation rate from pupae to adult in different containers	[0.08, 0.15] *day*^*−1*^	[26]
*ζ*	Amplitude of the seasonal variation in the reproduction rate of vectors	[0, 0.9]	[26]
*A*	Effect produced by temephos in the target household containers	0.03	[34, 35]
*k*_*max*_	Total maximum capacity of community participation effectiveness for the control of the dengue vector	0.13	[12]
*p*_*F*_	Proportion of participation of women in control mosquito activities	0.25	Value taken from the *Camino Verde* trial database
*p*_*M*_	Proportion of participation of men in control mosquito activities	0.02	Value taken from the *Camino Verde* trial database
*C*_*0*_	Effectiveness of pre-existing community participation	0.001	Proposed by the simulation of the logistic function
*r*	Incremental rate of community participation effectiveness	0.015	Proposed by the simulation of the logistic function

Since $M={\overline{S} }_{v}+{\overline{I} }_{v}$, we see from the last eight equations of (3) that mosquito dynamics from egg stage to adult form is given by

4 $$\begin{array}{l}\frac{dM}{dt}={\gamma }_{thc}{\left(t\right)P}_{thc}+{{\gamma }_{nthc}\left(t\right)P}_{nthc}-\left({\mu }_{v}+C\left(t\right)\right)M\\ \frac{d{E}_{thc}}{dt}={\theta }_{thc}\left(1-\frac{{E}_{thc}}{{E}_{thc}^{{\text{max}}}}\right)M-\left({\varepsilon }_{thc}+{\mu }_{E}+C\left(t\right)\right){E}_{thc}\\ \frac{d{E}_{nthc}}{dt}={\theta }_{nthc}\left(1-\frac{{E}_{nthc}}{{E}_{nthc}^{{\text{max}}}}\right)M-\left({\varepsilon }_{nthc}+{\mu }_{E}+C\left(t\right)\right){E}_{nthc}\\ \frac{d{L}_{thc}}{dt}={\varepsilon }_{thc}\left(1-\frac{{L}_{thc}}{{L}_{thc}^{{\text{max}}}}\right){E}_{thc}-\left({\lambda }_{thc}+{\mu }_{L}+A+C\left(t\right)\right){L}_{thc}\\ \frac{d{L}_{nthc}}{dt}={\varepsilon }_{nthc}\left(1-\frac{{L}_{nthc}}{{L}_{nthc}^{{\text{max}}}}\right){E}_{nthc}-\left({\lambda }_{nthc}+{\mu }_{L}+C\left(t\right)\right){L}_{nthc}\\ \frac{d{P}_{thc}}{dt}={\lambda }_{thc}{L}_{thc}-\left({\gamma }_{thc}\left(t\right)+{\mu }_{P}+C\left(t\right)\right){P}_{thc}\\ \frac{d{P}_{nthc}}{dt}={\lambda }_{nthc}{L}_{nthc}-\left({\gamma }_{nthc}\left(t\right)+{\mu }_{P}+C\left(t\right)\right){P}_{nthc}\end{array}$$

We consider that the maturation rates from pupae to adult in the different type of container, ${\gamma }_{thc}\left(t\right)$ and ${\gamma }_{nthc}\left(t\right)$, are modelled for a sinusoidal function with 1-year period. This assumption allows us to simulate the peaks dengue seasons observed in [16] with the periodic function proposed in [33]

5 $${\gamma }_{thc}\left(t\right)={\gamma }_{nthc}\left(t\right)={\gamma }_{0}\left(1+\zeta {\text{cos}}\left(\frac{2\pi }{365}t+\varphi \right)\right),$$

where ${\gamma }_{0}$ average reproduction maturation rate from pupae to adult in the different type of container, $\zeta$ is the amplitude of the seasonal variation in the reproduction rate of vectors, with $0\le \zeta <1$, and $\varphi$ is the phase angle to adjust the peak season for mosquitoes.

### Community participation effect

Recognizing that community participation intervenes in vector control differently than temephos [7, 36, 37], the model assumes that community participation affects: i) all stages of the mosquito life cycle, ii) both types of containers, with and without temephos, iii) reduces the load of infection by reducing the probability of human-mosquito transmission; and iv) that temephos only acts in the larval stage.

Based on the results obtained by Andersson et al. [12], we believe community participation affects infection risk slowly at the beginning of the intervention, as a critical mass knowledge and interest accumulates. Once people gain experience with their selected control strategies, participation has an increasing effect until it peaks, after which smaller changes maintain the impact. For that reason, we used a non-standard logistic function to analyze this pattern on reduction of pupae per person index.

One way to model different collaborative scenarios by sex for the effectiveness of community participation would be given by *C(t)*, the sum of female and male contribution in the intervention, modelled by non-standard logistic functions for each group, where *C*_*0*_ is the pre-existing effectiveness of community participation. Community participation is not a Boolean on/off switch. In its initial stages, it might be quite clumsy and inefficient. As participants experience the impact of their efforts, this gains momentum and effectiveness. *r* is the incremental rate of community participation effectiveness, modelling the idea that experiencing impact reinforces self-confidence and motivation [12]. The mobilisation strategy might show little effectiveness at the beginning of community participation, then increase in effectiveness is a function of r and finally stability of effectiveness after some time. If *k*_*max*_ is the total maximum capacity of community participation effectiveness for the control of the dengue vector, then the women’s contribution capacity, *k*_*F*_, is a proportion to this quantity, where *p*_*F*_ and *p*_*M*_are the proportions of participation of women and men in control mosquito activities While participation of both women and men is important, several studies have reported greater involvement of women than men in community dengue vector control [16, 38, 39]. The general case is therefore *p*_*F*_ > *p*_*M*_ (see S1 Appendix).

*C(t)* represents the maximum effectiveness of community participation at time *t* ∈ [*0,T*] of 13%, according to superior value of 95%CI for risk difference of pupae per person index calculated in Nicaragua and Mexico [12]. To achieve this risk difference reduction, about 25% of women reported participation in control vector activities, while only 2% of men participated in such activities (see S2 Appendix).

Temephos efficacy depends of different factors related to temephos doses, water turnover rate, type of water, and environmental factors around water storage such as organic debris presence, temperature and exposure to sunlight [8]. In studies reporting risk difference in the pupa per person index between 1 and 5%, the residual effect of temephos lasted from two and three months [34, 35]. Our model assume a constant temephos effect of 3%, as an average of the values found in the literature, which we believe balances out the effectiveness of the intervention during different seasons of the year.

Our model considers the effects of different levels of male and female participation. In this way the non-standard logistic function, *C(t)*, provides evidence of the effectiveness of community participation, as a strategy for the control of *Aedes aegypti*, considering the separate contributions of man and women.

### Analysis of the model

We define the net reproductive number of the mosquito population and then focus on the dynamics of dengue transmission applying a formula for the basic reproductive number and an estimate of the endemic equilibrium point.

### Mosquito dynamics

We analyse the mosquito population dynamics given by system (4). Considering constant values of the parameters, system (4) has two equilibrium points, the mosquitoes-free state *E*_*0*_ = (*0,0,0,0,0,0,0*) which correspond to the trivial equilibrium, and the state characterized by the presence of mosquitoes denoted by

9 $${E}_{1}=\left({M}^{*},{E}_{thc}^{*},{E}_{nthc}^{*},{L}_{thc}^{*},{L}_{nthc}^{*},{P}_{thc}^{*},{P}_{nthc}^{*}\right).$$

With the elements of *E*_*1*_ we obtained the basic offspring number, denote by *R*_*M*_ (details in S3 Appendix).

### The basic reproductive number

By analysing the dengue dynamics, we will assume that *R*_*M*_ > 1, since otherwise the mosquito population is zero, and therefore there is no disease. For *R*_*M*_ > 1, solutions approach asymptotically the equilibrium *E*_1_, and therefore we can assume that the mosquito population has already reached its equilibrium, and the total population of adult mosquitos is constant and equal to $M^{\ast}$. Considering that ${S}_{v}=\frac{{\overline{S} }_{v}}{{M}^{*}}$, ${I}_{v}=\frac{{\overline{I} }_{v}}{{M}^{*}}$ and *S*_*v*_ = 1-*I*_*v*_, model (3) is equivalent to the system of differential equations for the proportions

14 $$\begin{array}{l}\frac{d{S}_{h}}{dt}={\mu }_{h}\left(1-{S}_{h}\right)-\alpha {\beta }_{vh}\left(1-C\right)\frac{{M}^{*}}{{N}_{h}}{{I}_{v}S}_{h}\\ \frac{d{I}_{h}}{dt}=\alpha {\beta }_{vh}\left(1-C\right)\frac{{M}^{*}}{{N}_{h}}{{I}_{v}S}_{h}-\left(\rho +{\mu }_{h}\right){I}_{h}\\ \frac{d{I}_{v}}{dt}=\alpha {\beta }_{hv}\left(1-C\right)\left(1-{I}_{v}\right){I}_{h}-\left({\mu }_{v}+C\right){I}_{v}\end{array}$$

Our model hinges on the effective reproduction number of the disease, *R*_*eff*_, the number of people in a population who can be infected by an individual at any specific time (14). *R*_*eff*_ is a fraction of the basic reproduction number R_0_, the average number of secondary cases that one case can produce if introduced into a susceptible population of humans and mosquitoes. If R_0_ < 1, less than one secondary case will arise from a primary case and the disease will fade out. If R_0_ > 1 an outbreak will start.

The model has two equilibria, the disease-free state P_0_ = (1,0,0), and the dengue-present state denoted by

15 $${P}_{1}=\left(\frac{1}{\left(1-{I}_{v}^{*}\right){R}_{eff}},\frac{C+{\mu }_{v}}{\alpha {\beta }_{hv}\left(1-C\right)\left(1-{I}_{v}^{*}\right)}{I}_{v}^{*},{I}_{v}^{*}\right),$$

where

${I}_{v}^{*}=\frac{{\mu }_{h}{N}_{h}\left(C+{\mu }_{v}\right)\left(\rho +{\mu }_{h}\right)\left({R}_{eff}-1\right)}{\alpha {\beta }_{vh}\left(1-C\right){M}^{*}\left[\left(C+{\mu }_{v}\right)\left(\rho +{\mu }_{h}\right)+{\mu }_{h}\alpha {\beta }_{hv}\left(1-C\right)\right]}$ and *R*_eff_ is obtained from the follow lemma

*Lemma 3.2.1* Considered that the maturation rates from pupae to adult in the different type of container, *γ*_*thc*_(*t*) and *γ*_*nthc*_(*t*), and the effectiveness of community participation, *C*(*t*), are continuos, bounded, positive, periodic (in the case of *γ*_*thc*_(*t*)) and not identically zero functions of time. The average values ${\widehat{\gamma }}_{thc}\left(t\right)$ and $\widehat{C}(t)$ taken over a cycle are ${\widehat{\gamma }}_{thc}\left(t\right)=\frac{1}{t}{\int }_{0}^{t}{\gamma }_{thc}\left(x\right)dx$ and $\widehat{C}\left(t\right)=\frac{1}{t}{\int }_{0}^{t}C\left(x\right)dx$; if *γ*_*thc*_(*t*) and *C*(*t*) are replaced by that average values ${\widehat{\gamma }}_{thc}\left(t\right)$ and $\widehat{C}(t)$ then effective reproduction number of the disease is

16 $${R}_{eff}=\frac{{\widehat{M}}^{*}}{{N}_{h}}\frac{{\alpha }^{2}{\beta }_{hv}{\beta }_{vh}{\left(1-\widehat{C}\right)}^{2}}{\left(\widehat{C}+{\mu }_{v}\right)\left(\rho +{\mu }_{h}\right)}=\frac{{{{\mu }_{v}\widehat{M}}^{*}\left(1-\widehat{C}\right)}^{2}}{{\left(\widehat{C}+{\mu }_{v}\right)}N_{v}}\frac{{N}_{v}}{{N}_{h}}\frac{{\alpha }^{2}{\beta }_{hv}{\beta }_{vh}}{{\mu }_{v}\left(\rho +{\mu }_{h}\right)}=\frac{{{{\mu }_{v}\widehat{M}}^{*}\left(1-\widehat{C}\right)}^{2}}{\left(\widehat{C}+{\mu }_{v}\right){N}_{v}} {R}_{0}$$

where ${N}_{v}$ is the number of mosquitoes in the absence of intervention, ${R}_{0}$ is the basic reproduction number and ${\widehat{M}}^{*}$ is obtained according [40–42] to replaced ${\widehat{\gamma }}_{thc}$ and* Ĉ*(*t*). The average values ${\widehat{\gamma }}_{thc}\left(t\right)$ and *Ĉ*(*t*) are

$${\widehat{\gamma }}_{thc}\left(t\right)={\widehat{\gamma }}_{nthc}\left(t\right)={\gamma }_{0}+\frac{365}{2\pi t}\zeta {\gamma }_{0}\left(\left({\text{sin}}\left(\varphi \right){\text{cos}}\left(\frac{2\pi t}{365}\right)+{\text{sin}}\left(\frac{2\pi t}{365}\right){\text{cos}}\left(\varphi \right)\right)-{\text{sin}}\left(\varphi \right)\right)\mathrm{\, and\, }\widehat{C}\left(t\right)=\frac{1}{t}\left(\frac{{k}_{F}}{r}{\text{ln}}\left(1+\frac{{C}_{0}\left({e}^{rt}-1\right)}{{k}_{F}}\right)+\frac{\left({k}_{{\text{max}}}-{k}_{F}\right)}{r}{\text{ln}}\left(1+\frac{{C}_{0}\left({e}^{rt}-1\right)}{\left({k}_{{\text{max}}}-{k}_{F}\right)}\right)\right).$$

*Proof* From (5) is obtained that ${\gamma }_{thc}\left(t\right)={\gamma }_{0}\left(1+\zeta {\text{cos}}\left(\frac{2\pi }{365}t+\varphi \right)\right)$*,* then by definition of ${\widehat{\gamma }}_{thc}\left(t\right)$ is obtained that ${\widehat{\gamma }}_{thc}\left(t\right)=\frac{1}{t}{\int }_{0}^{t}{\gamma }_{0}\left(1+\zeta {\text{cos}}\left(\frac{2\pi }{365}t+\varphi \right)\right)dx$. Resolving the integral is obtained that ${\int }_{0}^{t}{\gamma }_{0}\left(1+\zeta {\text{cos}}\left(\frac{2\pi }{365}t+\varphi \right)\right)dx=\left(t{\gamma }_{0}+\frac{365}{2\pi }\zeta {\gamma }_{0}{\text{sin}}\left(\varphi +\frac{2\pi }{365}t\right)-\frac{365}{2\pi }\zeta {\gamma }_{0}{\text{sin}}\left(\varphi \right)\right)$, so

$${\widehat{\gamma }}_{thc}\left(t\right)={\gamma }_{0}+\frac{365}{2\pi t}\zeta {\gamma }_{0}\left(\left({\text{sin}}\left(\varphi \right){\text{cos}}\left(\frac{2\pi t}{365}\right)+{\text{sin}}\left(\frac{2\pi t}{365}\right){\text{cos}}\left(\varphi \right)\right)-{\text{sin}}\left(\varphi \right)\right).$$

If *C(t)* = *C*_*F*_*(t)* + *C*_*M*_*(t)*, then by definition *Ĉ*(*t*), and considering (6) and (7), getting $\widehat{C}\left(t\right)=\frac{1}{t}{\int }_{0}^{t}\left[\frac{{k}_{F}{C}_{0}{e}^{rt}}{{k}_{F}+{C}_{0}\left({e}^{rt}-1\right)}+\frac{\left({{k}_{{\text{max}}}-k}_{F}\right){C}_{0}{e}^{rt}}{\left({{k}_{{\text{max}}}-k}_{F}\right)+{C}_{0}\left({e}^{rt}-1\right)}\right]dx$. Resolving and simplifying ${\int }_{0}^{t}\left(\frac{{k}_{F}{C}_{0}{e}^{rt}}{{k}_{F}+{C}_{0}\left({e}^{rt}-1\right)}+\frac{\left({{k}_{{\text{max}}}-k}_{F}\right){C}_{0}{e}^{rt}}{\left({{k}_{{\text{max}}}-k}_{F}\right)+{C}_{0}\left({e}^{rt}-1\right)}\right)dx=\left(\frac{{k}_{F}}{r}{\text{ln}}\left(1+\frac{{C}_{0}\left({e}^{rt}-1\right)}{{k}_{F}}\right)+\frac{\left({k}_{{\text{max}}}-{k}_{F}\right)}{r}{\text{ln}}\left(1+\frac{{C}_{0}\left({e}^{rt}-1\right)}{\left({k}_{{\text{max}}}-{k}_{F}\right)}\right)\right)$, from where

$$\widehat{C}\left(t\right)=\frac{1}{t}\left(\frac{{k}_{F}}{r}{\text{ln}}\left(1+\frac{{C}_{0}\left({e}^{rt}-1\right)}{{k}_{F}}\right)+\frac{\left({k}_{{\text{max}}}-{k}_{F}\right)}{r}{\text{ln}}\left(1+\frac{{C}_{0}\left({e}^{rt}-1\right)}{\left({k}_{{\text{max}}}-{k}_{F}\right)}\right)\right).$$

### Estimation on the human population for Acapulco city

In the last century, the Acapulco’s population size had a non-linear increase [43] though, from the year 2000, the increase was more stable. We therefore adjusted a non-linear regression model to the data of the Acapulco population between the years 1910–2020. The non-linear function considered was as follows:

$$y=\frac{K{u}_{0}}{{u}_{0}+\left(K-{u}_{0}\right){e}^{-\mathcal{R}x}},$$

where *y* is the Acapulco population, *x* is time in years, *u*_*0*_ is the initial population, *K* the saturation capacity and *R* the population growth rate. A value of 834 634 was obtained as a estimator of *K* with a 95% CI [803 423.7, 865 844.3], used as the population size of Acapulco *N*_*h*_, and is assumed approximately constant in the next five years.

## Results

### Simulation of effectiveness of community participation

**Fig. 2:**
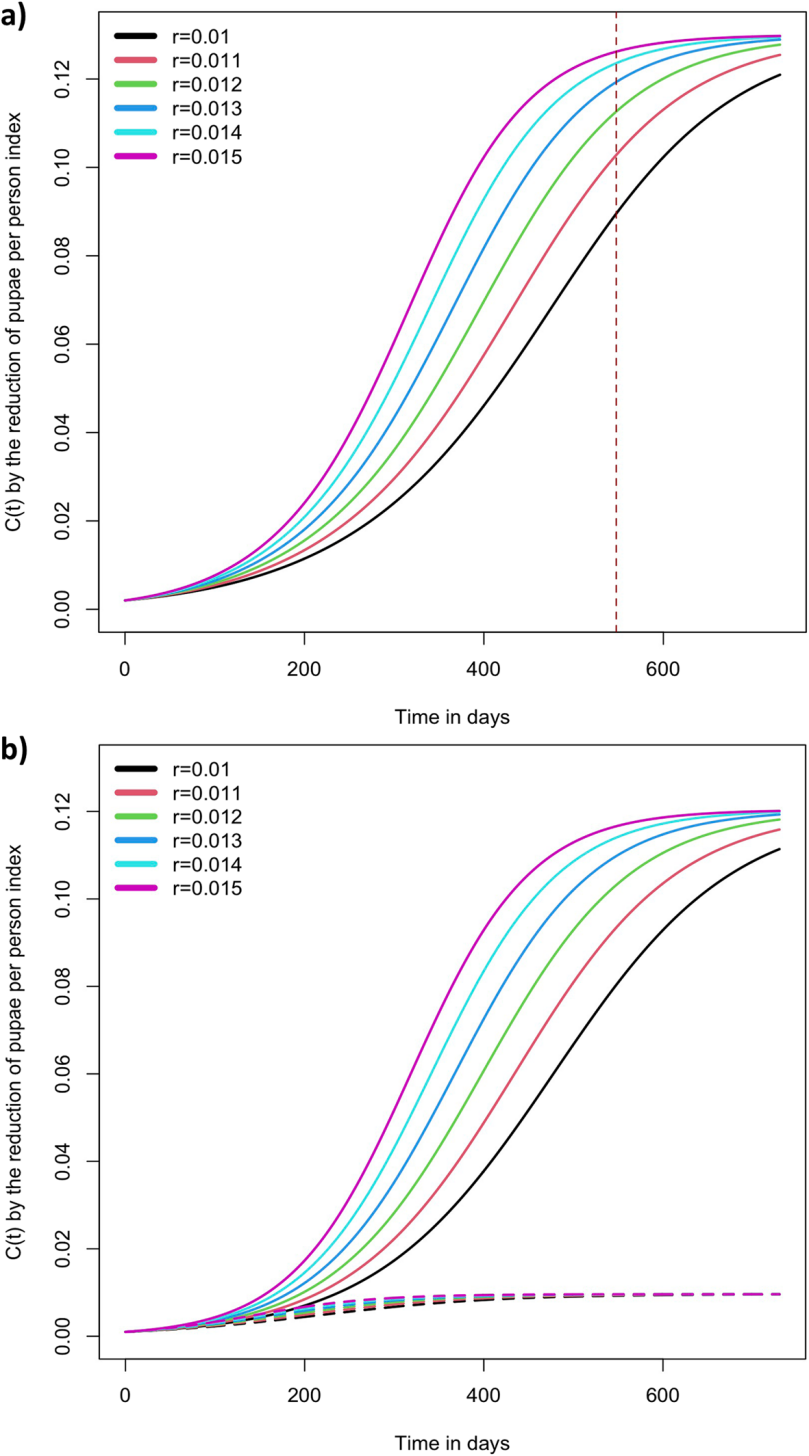
Effectiveness of community participation represented by the reduction of pupae per person index modelled by logistical function. **a** Overall effectiveness of community participation, **b** Effectiveness of community participation by gender (solid lines represents women results, dotted lines men)

Figure 2 shows the modelling of total effectiveness of community participation. This considers a strategy aimed to trigger community participation achieves the greatest effectiveness in one and a half years (dotted line in panel ‘a’ of Fig. 2), and an estimator for incremental rate of community participation effectiveness is *r* = 0.015. Panel ‘b’ shows the community participation effectiveness divided by gender, dotted curves is the contribution of men for different *r* values.

The basic reproductive number for dengue cases is significantly affected by community participation.

**Fig. 3:**
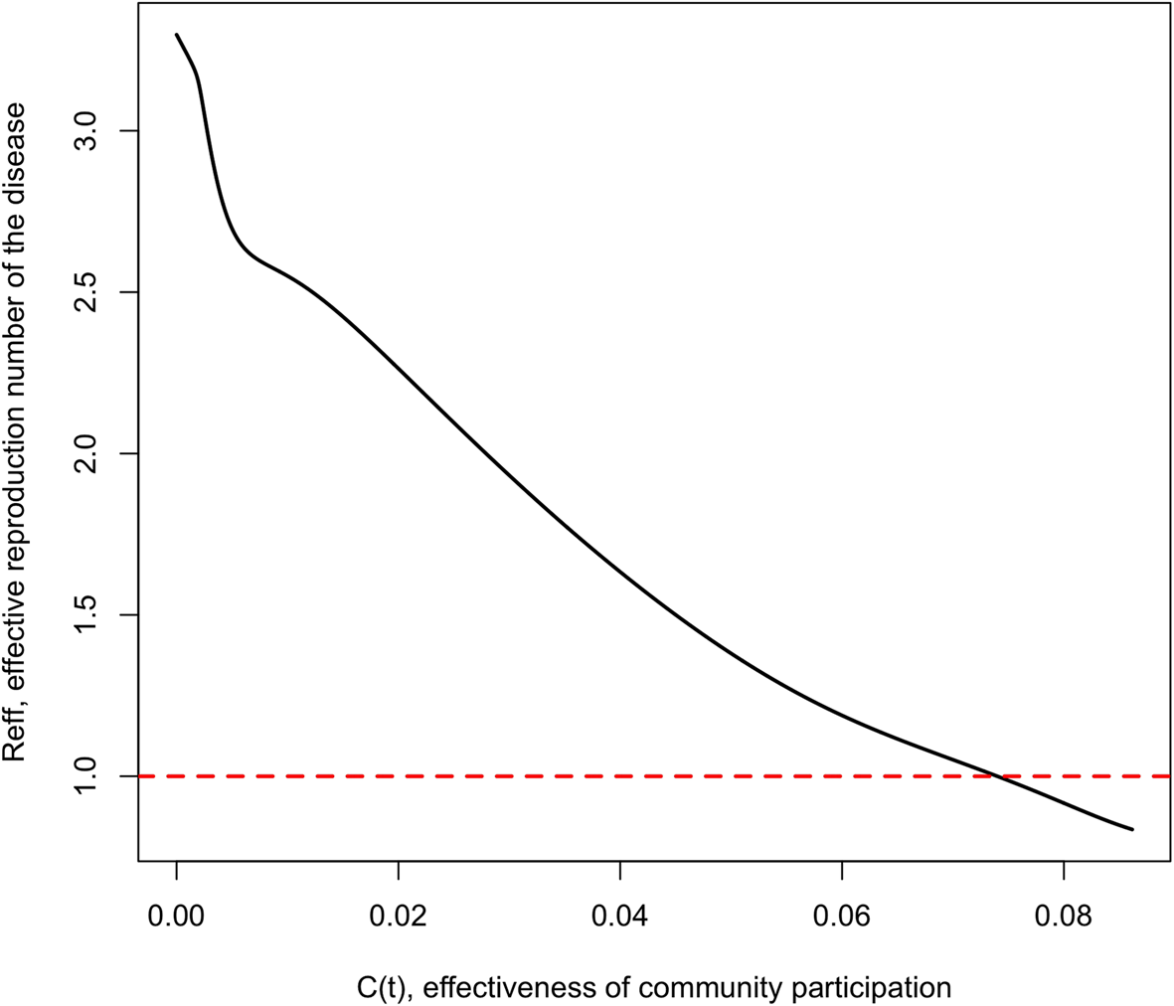
Variation of the effective reproduction number *R*_*eff*_ with respect effectiveness of community participation. For *Ĉ*(*t*) in *t* ∈ [0,*T*], ${\widehat{\gamma }}_{thc}\left(t\right)$ and ${\widehat{\gamma }}_{nthc}\left(t\right)$, with *p*_*F*_ = 0.25, *p*_*M*_ = 0.02, *C*_*0*_ = 0.001, *r* = 0.015, *ζ* = 0.5 and *φ* = π/2

Taking the maximum values of E_thc_, E_nthc_, L_thc_ and L_nthc_ when the populations of eggs and larvae stabilize over time, then when no intervention is implemented on *Aedes aegyti* control, the obtained value is *R*_*eff*_ = *R*_*0*_ = 3.3. If an intervention with temephos is implemented, considering a constant effectiveness of 3%, the obtained value is *R*_*eff*_ = 3.1, both scenarios are auspicious for the occurrence of a dengue outbreak. When social mobilization achieves an effectiveness of 8.5%, as result of community participation added to temephos intervention, the value obtained is *R*_*eff*_ < 1 (Fig. 3). Under these circumstances, control of dengue cases is achieved.

### Simulation of dynamic of dengue

The simulation was carried out using the values of Table 1 and the initial values ${\overline{S} }_{h}\left(0\right)=833799$, ${\overline{I} }_{h}\left(0\right)=835$, *R*_*h*_(0) = 0 in addition *E*_*1*_ = (974666, 866743, 866743, 317083, 317083, 487821, 487821) give by (9). We implemented the simulation using the “deSolve” package of the R [44].

**Fig. 4:**
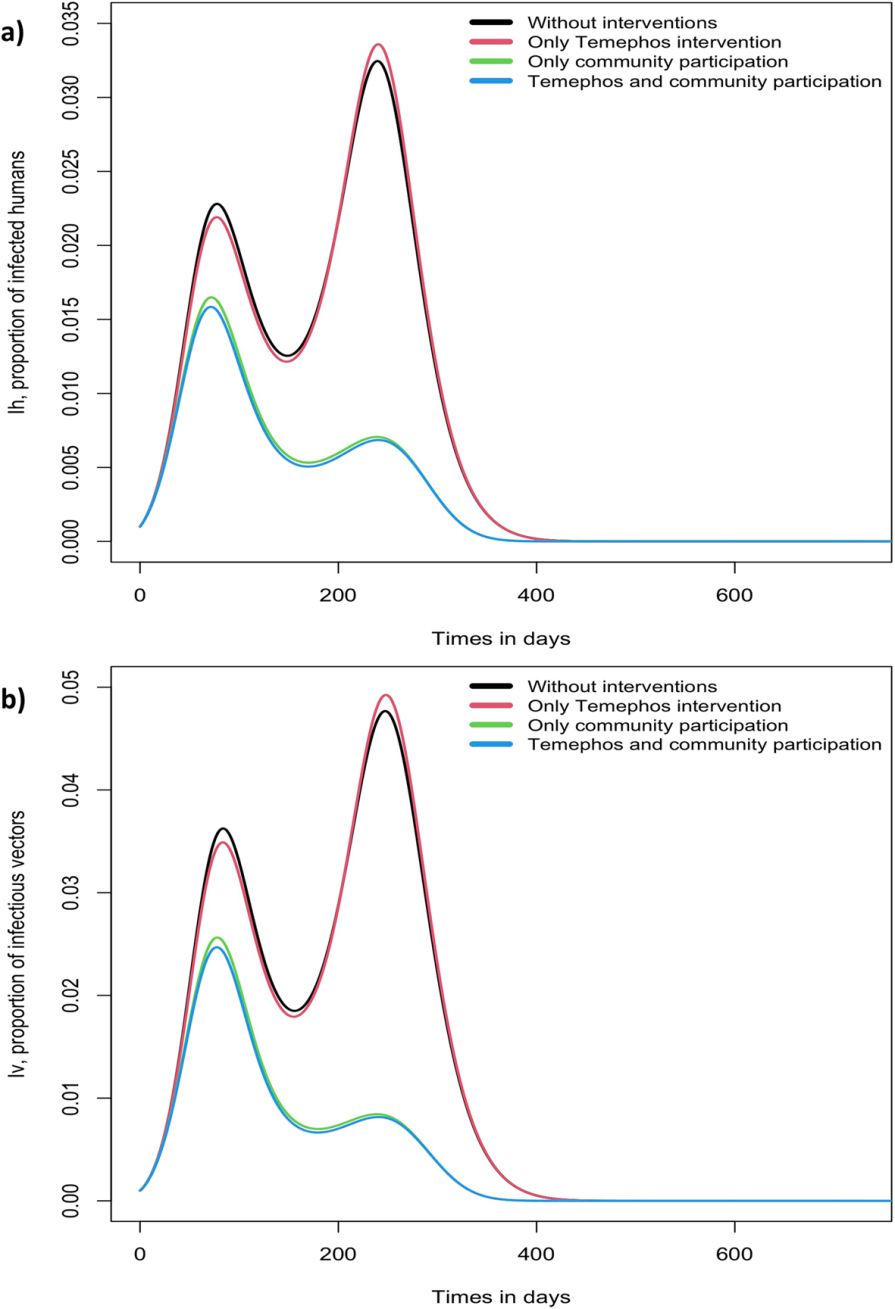
Proportions of infected humans and infectious mosquitos for different types of interventions against *Aedes aegypti*

Figure 4 shows the proportions of infected humans and infectious vectors from the simulation model (3). Up to forty days after the start of the dengue outbreak, the different interventions for *Aedes aegypti* control produce the same results. There is no evidence of protective effect of temephos intervention 220 days after outbreak initiation: the greater effect in short term - in terms of people infected with dengue - was the combination of temephos intervention and community participation (Fig. 4).

### Simulation considering different levels of community participation by gender

**Fig. 5:**
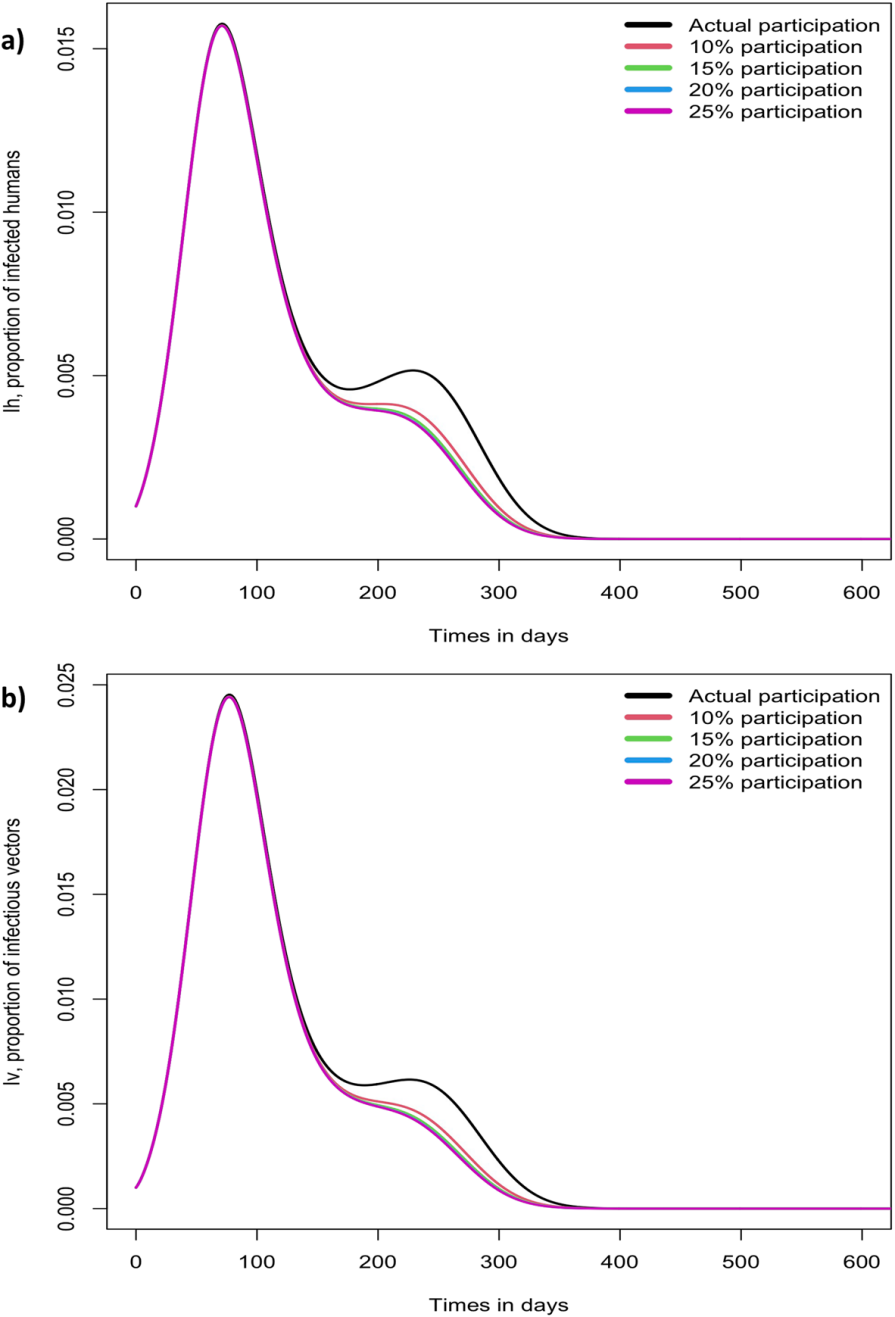
Proportions of infected humans and infective mosquitoes with different scenarios of men contribution to community participation

Figure 5 shows different scenarios of the proportions of infected people and infectious mosquitoes. The simulation showed how increasing the contribution of men in community participation, to its initial participation of 2% and gradually increasing up to 25%, decreases more rapidly the proportion of infected people and infectious mosquitoes after day 120, it reaches its maximum difference 250 days after the start of the outbreak; in this period, there was no significant difference in the proportions of infected people and infectious mosquitoes, even when the percentage of participation of men increased by more than 10% (Fig. 5).

## Discussion

Our model is well defined biologically and mathematically. It suggests that, in the long term, community participation on its own produces the strongest protection. Adding temephos to community participation does not improve its effect, possibly because temephos might produce a false feeling protection [15]. Our compartmental model is compatible with the association reported by Andersson et al. [12] between use of temephos and increased risk of dengue virus infection. The *Camino Verde* trial showed a 13% reduction in the pupal index per person with community engagement, with higher levels of protection in clusters where women engaged in vector control activities.

Our simulation of dengue endemicity reflects a basic reproductive number significantly affected by community participation. An initial reduction associated with temephos use can be explained by the combination of temephos intervention and community participation (Fig. 4). There was no protective effect of temephos intervention 220 days after the outbreak began, this agrees with the Nicaraguan report, where exposure to temephos was associated with higher entomological indices over time [40]. This was explained by several factors including the ecological adaptability of the vector, resistance of Aedes to the pesticide, operational deficiencies of the vector control program (lower quantities used at greater intervals in only a sample of reservoirs), a decrease in community motivation as a consequence of a false sense of protection when temephos is present in their water. Our simulation showed increasing the contribution of men in community participation by more than 10% might decrease slightly the proportions of infected people and infectious mosquitoes (Fig. 5).

Community participation affects the risk of infection in a variable way. Initially there may be little change. As a critical mass of interest, knowledge and confidence accumulates, the quality of participation changes and its effect is more noticeable. After people gain proficiency with a particular control strategy, implementing it fully, the biological potential of the specific activity to control reproduction reaches a peak, before adaptation of the vector mosquito.

We did simulations that calculate *R*_*0*_ for the different strategies, taking the maximum values of E_thc_, E_nthc_, L_thc_ and L_nthc_ when the populations of eggs and larvae stabilize over time. This generates an effectiveness of community participation of 8.5%, translating as a reduction of *R*_*0*_ < 1. The model assumes a constant biting rate but reduced human-vector contact, with a transmission rate $\alpha {\beta }_{vh}\left(1-C\left(t\right)\right){\overline{S} }_{h}\frac{{\overline{I} }_{v}}{{N}_{h}}$, where $1-C\left(t\right)$ describes the reduction in contacts between infected mosquitos and susceptible humans, reflecting the reduced mosquito population. This approach has been used in other models of vector-borne diseases [25, 26, 45].

Mathematical models have proved useful to understand the transmission of dengue and to help plan infection control strategies [17–29]. Optimizing the intervention mix of control strategies is a relatively recent trend in mathematical modelling. Our compartmental model, without identifying an optimum control approach, helps to explain transmission dynamics according to the reduction of the relative risk for the proportion of infected people and the rate of pupae per person.

### Limitations

Our model does not consider the variations in the environment (temperature, humidity and height above sea level), and community location (urban, rural or suburbs) which are important if partially studied determinants of the dengue vector density. [29, 46] Most of Acapulco is less than 300 m above sea level, the environmental temperature is warm most of the year, and humidity differs depending with the rainy season. Our model took the variation in humidity into account by a sinusoidal function for estimating the pupal population. Other limitation is the lack of another compartment, in this specific case, the estimated population into latent period of the disease, which is not included in the SIR model.

Dengue mortality occurs mainly in children and the elderly and fortunately there is a low mortality rate due to severe dengue. We did not include the effect of mortality on transmission. We based simulations on the Acapulco general population, so not including mortality indicators will have little effect on the model.

Our lack of longitudinal data on infected individuals in intervention and control sites limits our ability to inform our model with actual dengue cases in the trial setting. We used historical dengue case reports from Acapulco. Both 2013 and 2015 reported two outbreaks in the year, the second with a higher incidence than the first. Shepard et al. [47] suggested an expansion factor of 1.4–3.3 to allow for the well-recognized under-reporting. The results of our simulation without the intervention thus coincide with 2–3 times the average number of cases reported during the peaks of the outbreaks in Acapulco in the years preceding the trial, 7 307 in 2013 and 5 586 in 2015.

We considered the temephos effect without any variation in effectiveness or use between households. Temephos effectiveness varies dramatically with the dose present and amount of water in the container, mosquito breeding density, time temephos is left in containers, rain season and other factors. Our model used average temephos effectiveness, possibly overestimating its impact without affecting the results of community participation. It was possible to consider the time dependence of the effect of temephos, as the Fig. 4 shows, after 220 days effectiveness of temephos dropped drastically.

We did not model different engagement dynamics for men and women [16]. We are aware of different roles in participatory vector control – men in Mexico would be more concerned with outdoor or community actions, with women more involved in household activities. We modelled the effect as additive, although other interactions (synergism or proportional functions) may have played a role. This aspect of our model could hide the real effect of gender in community participation not least, as pointed out by Andersson and colleagues [12], the possibility that the way *Camino Verde* engaged women acted as a disincentive to male engagement. There could be unidentified ripple effects of more equitable engagement, including better family communication, reduction of the women workload.

Community participation adds value to conventional control strategies for *Aedes aegypti* control for dengue fever. Our model suggests that, in the long term, use of temephos alone could fail to achieve *Aedes aegypti* control, whereas community participation might have a more sustained effect.

### Supplementary Information

**Supplementary Material 1.**

**Supplementary Material 2.**

**Supplementary Material 3.**

## Data Availability

The datasets used and/or analysed during the current study are available from the corresponding author on reasonable request.

## Abbreviations

S1: Supplement 1
S2: Supplement 2
S3: Supplement 3
SIR: Susceptible-infected-recovered model

## References

[1] World Health Organization. Dengue and severe dengue. http://www.who.int/mediacentre/factsheets/fs117/en/. Accessed 15 June 2022.

[2] Pan American Health Organization, World Health Organization. Scientists studying intensified vector control measures to combat Zika, dengue and chikungunya in the Americas. http://www.paho.org/hq/index.php?option=com_content&view=article&id=11780. Accessed 15 June 2022.

[3] Bhatt S (2013). The global distribution and burden of dengue. Nature.

[4] Hayes EB (2009). Zika virus outside Africa. Emerg Infect Dis.

[5] Montero A (2015). Chikungunya fever – a new global threat. Med Clin.

[6] Gill GV, Beeching N (2014). Tropical medicine: lecture notes.

[7] World Health Organization. Dengue: guidelines for diagnosis, treatment, prevention and control. http://whqlibdoc.who.int/publications/2009/9789241547871_eng.pdf. Accessed 17 June 2022. 23762963

[8] George L (2015). Community-effectiveness of temephos for dengue vector control: a systematic literature review. PLoS Negl Trop Dis.

[9] Garza-Robledo AA (2011). Effectiveness of Spinosad and Temephos for the control of mosquito larvae at a tire dump In Allende, Nuevo Leon, Mexico. J Am Mosq Control Assoc.

[10] Grisales N (2013). Temephos resistance in *Aedes aegypti* in Colombia compromises dengue vector control. PLoS Negl Trop Dis.

[11] Ocampo CB (2011). Insecticide resistance status of *Aedes aegypti* in 10 localities in Colombia. Acta Trop.

[12] Andersson N (2015). Evidence based community mobilization for dengue prevention in Nicaragua and Mexico (*Camino Verde*, the Green Way): cluster randomized controlled trial. BMJ.

[13] Heintze C, Velazco Garrido M, Kroeger A (2007). What do community-based dengue control programmes achieve? A systematic review of published evaluations. Trans R Soc Trop Med Hyg.

[14] Erlanger TE, Keiser J, Utzinger J (2008). Effect of dengue vector control interventions on entomological parameters in developing countries: a systematic review and meta-analysis. Med Vet Entomol.

[15] Alvarado-Castro V (2017). Assessing the effects of interventions for *Aedes aegypti* control: systematic review and meta-analysis of cluster randomised controlled trials. BMC Public Health.

[16] Andersson N (2017). The women made it work: fuzzy transitive closure of the results chain in a dengue prevention trial in Mexico. BMC Public Health.

[17] Esteva L, Vargas C (1998). Analysis of a dengue fever disease transmission model. Math Biosci.

[18] Esteva L, Vargas C (1999). A model for dengue disease with variable human population. J Math Biol.

[19] Garba SM, Gumel AB, Bakar MR (2008). Backward bifurcations in dengue transmission dynamics. Math Biosci.

[20] Li-Martín A, Reyes-Carreto R, Vargas-De-León C (2023). Dynamics of a dengue disease transmission model with two-stage structure in the human population. Math Biosci Eng.

[21] Predescu M (2007). On the dynamics of a deterministic and stochastic model for mosquito control. Appl Math Lett.

[22] Burattini MN (2008). Modelling the control strategies against dengue in Singapore. Epidemiol Infect.

[23] Abidemi A, Abd-Aziz M, Ahmad R (2020). Vaccination and vector control effect on dengue virus transmission dynamics: modelling and simulation. Chaos Solit Fractals.

[24] Abboubakar H, Kamgang JC, Tieudjo D (2016). Backward bifurcation and control in transmission dynamics of arboviral diseases. Math Biosci.

[25] Buonomo B, Della MR (2018). Optimal bed net use for a dengue disease model with mosquito seasonal pattern. Math Methods Appl Sci.

[26] Asamoah JKK (2021). Optimal control and cost-effectiveness analysis for dengue fever model with asymptomatic and partial immune individuals. Results Phys.

[27] Naidu DS (2002). Optimal control systems.

[28] Ndekha A (2003). Community participation as an interactive learning process: experiences from a schistosomiasis control project in Zimbabwe. Acta Trop.

[29] Esteva L, Yang HM (2015). Assessing the effects of temperature and dengue virus load on dengue transmission. J Biol Syst.

[30] Ai S, Li J, Lu J (2012). Mosquito-stage-structured malaria models and their global dynamics. SIAM J Appl Math.

[31] Moulay D, Aziz-Alaoui MA, Cadivel M (2011). The chikungunya disease: modeling, vector and transmission global dynamics. Math Biosci.

[32] Consejo Nacional de Población, México. Documento metodológico: proyecciones de la Población 2010–2050. 2012. http://www.conapo.gob.mx/work/models/CONAPO/Resource/1529/2/images/DocumentoMetodologicoProyecciones2010_2050.pdf. Accessed 2 July 2022.

[33] Moulay D, Azizz-Alaoui MA, Kwon H (2012). Optimal control of chikungunya disease: larvae reduction, treatment and prevention. Math Biosci Eng.

[34] Chadee DD (2009). Impact of pre-seasonal focal treatment on population densities of the mosquito *Aedes aegypti* in Trinidad, West Indies: a preliminary study. Acta Trop.

[35] Morales-Pérez A (2017). *Aedes aegypti* breeding ecology in Guerrero: cross-sectional study of mosquito breeding sites from the baseline for the Camino Verde trial in Mexico. BMC Public Health.

[36] Sztankay-Gulyás M (1972). Mosquito control with integrated method. Wiad Parazytol.

[37] Saide PM (2019). Technical document for the implementation of interventions based on generic operational scenarios for *Aedes aegypti* control.

[38] Gunn (2018). Current strategies and successes in engaging women in vector control: a systematic review. BMJ Glob Health.

[39] Mungall-Baldwin C (2022). Women’s participation in the prevention and control of dengue using environmental methods in the global south: a qualitative meta-synthesis. Int J Equity Health.

[40] Arosteguí J (2017). Beyond efficacy in water containers: temephos and household entomological indices in six studies between 2005 and 2013 in Managua, Nicaragua. BMC Public Health.

[41] Chowell G (2008). Spatial and temporal dynamics of dengue fever in Peru: 1994–2006. Epidemiol Infect.

[42] Chowell G (2007). Estimation of the reproduction number of dengue fever from spatial epidemic data. Math Biosci.

[43] National institute of statistics and geography, INEGI. Population and housing census and counting. http://en.www.inegi.org.mx/programas/ccpv/1900/. Accessed 24 June 2022.

[44] Soetaert K, Petzoldt T, Setzer RW (2010). Solving differential equations in R: package deSolve. J Stat Softw.

[45] Abidemi A (2022). Lyapunov stability analysis and optimization measures for a dengue disease transmission model. Physica A.

[46] Mincham G (2019). Development of a mechanistic dengue simulation model for Guangzhou. Epidemiol Infect.

[47] Shepard DS (2011). Economic impact of dengue illness in the Americas. Am J Trop Med Hyg.

