# Supplementary material for: The influence of gender and temephos exposure on community participation in dengue prevention: a compartmental mathematical model
Source: BMC Infect Dis. 2024 May 2;24:463. doi: 10.1186/s12879-024-09341-w (PMC11067291; doi:10.1186/s12879-024-09341-w)
Supplement: Supplementary file 1 — Supplementary Material 1. [file 12879_2024_9341_MOESM1_ESM.docx]

**S1 Appendix. Modelled community participation.**

One way to model different collaborative scenarios by sex for the effectiveness of community participation would be given by *C*(*t*)=*C_F_*(*t*)+*C_M_*(*t*), where

$C_{F}\left( t \right)=\frac{k_{F}C_{0}e^{rt}}{k_{F}+C_{0}\left( e^{rt}-1 \right)}$, (6)

$C_{M}\left( t \right)=\frac{\left( {k_{\max}-k}_{F} \right)C_{0}e^{rt}}{\left( {k_{\max}-k}_{F} \right)+C_{0}\left( e^{rt}-1 \right)}$, (7)

where *k_max_* is the total maximum capacity of community participation effectiveness for the control of the dengue vector, *k_F_* is the women’s contribution capacity modelled by

$k_{F}=\frac{p_{F}}{p_{F}+p_{M}}k_{max}$, (8)

where *p_F_* and *p_M_* are the proportions of participation of women and men in control mosquito activities, considering [16,38,39] it is assumed that pF> pM so that the contribution of women is greater than that of men.

*k_max_*−*k_F_* is the men’s contribution capacity; *C_0_* is the pre-existing effectiveness of community participation and *r* is the incremental rate of community participation effectiveness.
