# Supplementary material for: The influence of gender and temephos exposure on community participation in dengue prevention: a compartmental mathematical model
Source: BMC Infect Dis. 2024 May 2;24:463. doi: 10.1186/s12879-024-09341-w (PMC11067291; doi:10.1186/s12879-024-09341-w)
Supplement: Supplementary file 2 — Supplementary Material 2. [file 12879_2024_9341_MOESM2_ESM.docx]

**S2 Appendix. Participation by gender according to the Camino Verde trial database**

**Women and men stratified by intervention group**

|  | Intervention | Control | Total |
| --- | --- | --- | --- |
| Women | 3977 | 3923 | 7900 |
| Men | 3478 | 3285 | 6763 |
| Total | 7455 | 7208 | 14663 |

**Women and men who participated**

|  | Participation | No participation | Total |
| --- | --- | --- | --- |
| Women | 3420 | 4480 | 7900 |
| Men | 341 | 6422 | 6763 |
| Total | 3761 | 10902 | 14663 |

**Women and men who participated in the intervention group**

|  | Participation | No participation | Total |
| --- | --- | --- | --- |
| Women | 1861 | 2116 | 3977 |
| Men | 165 | 3313 | 3478 |
| Total | 2026 | 5429 | 7455 |

**Women and men who participated in the control group**

|  | Participation | No participation | Total |
| --- | --- | --- | --- |
| Women | 1559 | 2364 | 3923 |
| Men | 176 | 3109 | 3285 |
| Total | 1735 | 5473 | 7208 |
