# Supplementary material for: The influence of gender and temephos exposure on community participation in dengue prevention: a compartmental mathematical model
Source: BMC Infect Dis. 2024 May 2;24:463. doi: 10.1186/s12879-024-09341-w (PMC11067291; doi:10.1186/s12879-024-09341-w)
Supplement: Supplementary file 3 — Supplementary Material 3. [file 12879_2024_9341_MOESM3_ESM.docx]

**S3 Appendix. Obtained the basic offspring number** $\boldsymbol{R}_{\boldsymbol{M}}$**.**

Considering the equilibrium point, the state characterized by the presence of mosquitoes denoted by

$E_{1}=\left( M^{*},E_{thc}^{*},E_{nthc}^{*},L_{thc}^{*},L_{nthc}^{*},P_{thc}^{*},P_{nthc}^{*} \right)$, (9)

where

$P_{thc}^{*}=\frac{\lambda_{thc}}{\left( \gamma_{thc}+C+\mu_{P} \right)}L_{thc}^{*}$,

$P_{nthc}^{*}=\frac{\lambda_{nthc}}{\left( \gamma_{nthc}+C+\mu_{P} \right)}L_{nthc}^{*}$,

$E_{thc}^{*}=\frac{\theta_{thc}}{\frac{\theta_{thc}}{E_{thc}^{\max}}M^{*}+\left( \varepsilon_{thc}+C+\mu_{E} \right)}M^{*}$,

$E_{nthc}^{*}=\frac{\theta_{nthc}}{\frac{\theta_{nthc}}{E_{nthc}^{\max}}M^{*}+\left( \varepsilon_{nthc}+C+\mu_{E} \right)}M^{*}$, (10)

$L_{thc}^{*}=\frac{{\varepsilon_{thc}\theta}_{thc}}{\frac{{\varepsilon_{thc}\theta}_{thc}}{L_{thc}^{\max}}M^{*}+\left( \lambda_{thc}+C+A+\mu_{L} \right)\left( \frac{\theta_{thc}}{E_{thc}^{\max}}M^{*}+\left( \varepsilon_{thc}+C+\mu_{E} \right) \right)}M^{*}$,

$L_{nthc}^{*}=\frac{{\varepsilon_{nthc}\theta}_{nthc}}{\frac{{\varepsilon_{nthc}\theta}_{nthc}}{L_{nthc}^{\max}}M^{*}+\left( \lambda_{nthc}+C+\mu_{L} \right)\left( \frac{\theta_{nthc}}{E_{nthc}^{\max}}M^{*}+\left( \varepsilon_{nthc}+C+\mu_{E} \right) \right)}M^{*}$,

and *M^*^* which satisfies the quadratic equation *A*_1_*M*^2^+*A*_2_*M*−*A*_3_=0, where

$A_{1}=\frac{\left( \varepsilon_{nthc}E_{nthc}^{\max}+\left( \lambda_{nthc}+C+\mu_{L} \right)L_{nthc}^{\max} \right)\left( \varepsilon_{thc}E_{thc}^{\max}+\left( \lambda_{thc}+C+A+\mu_{L} \right)L_{thc}^{\max} \right)}{L_{nthc}^{\max}E_{nthc}^{\max}L_{thc}^{\max}E_{thc}^{\max}}\left( C+\mu_{v} \right)\theta_{thc}\theta_{nthc}$>0, $A_{2}=\left( \left( \lambda_{nthc}+C+\mu_{L} \right)\left( \varepsilon_{nthc}+C+\mu_{E} \right)\left( C+\mu_{v} \right)-\frac{\gamma_{nthc}\lambda_{nthc}\varepsilon_{nthc}\theta_{nthc}}{\gamma_{nthc}+C+\mu_{P}} \right)\left( \frac{\varepsilon_{thc}}{L_{thc}^{\max}}+\frac{\lambda_{thc}+C+A+\mu_{L}}{E_{thc}^{\max}} \right)\theta_{thc}+\left( \left( \lambda_{thc}+C+A+\mu_{L} \right)\left( \varepsilon_{thc}+C+\mu_{E} \right)\left( C+\mu_{v} \right)-\frac{\gamma_{thc}\lambda_{thc}\varepsilon_{thc}\theta_{thc}}{\gamma_{thc}+C+\mu_{P}} \right)\left( \frac{\varepsilon_{nthc}}{L_{nthc}^{\max}}+\frac{\lambda_{nthc}+C+\mu_{L}}{E_{nthc}^{\max}} \right)\theta_{nthc}$> 0 and $A_{3}=\left( C+\mu_{v} \right)\left( \lambda_{thc}+C+A+\mu_{L} \right)\left( \varepsilon_{thc}+C+\mu_{E} \right)\left( \lambda_{nthc}+C+\mu_{L} \right)\left( \varepsilon_{nthc}+C+\mu_{E} \right)\left[ 1-R_{M} \right]$. That is,

$M^{*}=\frac{-A_{2}+\left( A_{2}^{2}-4A_{1}A_{3} \right)^{1/2}}{2A_{1}}$. (11)

One can see that *A*_3_<0 for *R_M_*>1, *A*_3_=0 with *R_M_*=1 and *A*_3_>0 for *R_M_*<1. Hence, the quadratic equation has a positive root for all *R_M_*>1 and no positive root when *R_M_*<1.

The $R_{M}=R_{M}^{thc}+R_{M}^{nthc}$ denotes the basic offspring number where

$R_{M}^{thc}=\frac{\gamma_{thc}\lambda_{thc}\varepsilon_{thc}\theta_{thc}}{\left( C+\mu_{v} \right)\left( \lambda_{thc}+C+A+\mu_{L} \right)\left( \varepsilon_{thc}+C+\mu_{E} \right)\left( \gamma_{thc}+C+\mu_{P} \right)}$ (12)

and

$R_{M}^{nthc}=\frac{\gamma_{nthc}\lambda_{nthc}\varepsilon_{nthc}\theta_{nthc}}{\left( C+\mu_{v} \right)\left( \lambda_{nthc}+C+\mu_{L} \right)\left( \varepsilon_{nthc}+C+\mu_{E} \right)\left( \gamma_{nthc}+C+\mu_{P} \right)}$. (13)
